# Tomato (*Solanum lycopersicum* L.) accumulation and allergenicity in response to nickel stress

**DOI:** 10.1038/s41598-022-09107-x

**Published:** 2022-03-31

**Authors:** Enrica Roccotiello, Elena Nicosia, Lorenzo Pierdonà, Pietro Marescotti, Maria Antonietta Ciardiello, Ivana Giangrieco, Adriano Mari, Danila Zennaro, Denise Dozza, Michele Brancucci, Mauro Mariotti

**Affiliations:** 1 Department of Earth Environment and Life Sciences (DISTAV), Università degli Studi di Genova, Corso Europa 26, 16132 Genoa, Italy; 2 Regione Liguria, Dipartimento Salute e Servizi Sociali, Settore Tutela della Salute negli Ambienti di Vita e di Lavoro Via Fieschi 17, Piano U8, 16121 Genoa, Italy; 3 Department of Agroenvironmental Chemistry and Plant Nutrition, Czech University of Life Sciences, Kamýcká 129, Suchdol, 16500 Prague, Czech Republic; 4 Institute of Biosciences and BioResources (IBBR), CNR, Naples, Italy; 5 Allergy Data Laboratories (ADL), Latina, Italy; 6 Associated Centers for Molecular Allergology (CAAM), Rome, Italy; 7 IREN Laboratori S.P.a, Via SS. Giacomo E Filippo 7, 16122 Genoa, Italy; 8 Geospectra S.R.L. Spin Off, Via Palmaria 9/6, 16121 Genoa, Italy

**Keywords:** Metals, Abiotic, Plant sciences, Plant molecular biology, Plant stress responses, Proteins

## Abstract

Vegetables represent a major source of Ni exposure. Environmental contamination and cultural practices can increase Ni amount in tomato posing significant risk for human health. This work assesses the tomato (*Solanum lycopersicum* L.) response to Ni on the agronomic yield of fruits and the related production of allergens. Two cultivars were grown in pots amended with Ni 0, 30, 60, 120, and 300 mg kg^−1^, respectively. XRF and ICP-MS analyses highlighted the direct increase of fruit Ni content compared to soil Ni, maintaining a stable biomass. Leaf water content increased at Ni 300 mg kg^−1^. Total protein content and individual allergenic components were investigated using biochemical (RP-HPLC and N-terminal amino acid sequencing) and immunological (inhibition tests of IgE binding by SPHIAa assay on the FABER testing system) methodologies. Ni affected the fruit tissue concentration of pathogenesis-related proteins and relevant allergens (LTP, profilin, Bet v 1-like protein and TLP). This study elucidates for the first time that tomato reacts to exogenous Ni, uptaking the metal while changing its allergenic profiles, with potential double increasing of exposure risks for consumers. This evidence highlighted the importance of adequate choice of low-Ni tomato cultivars and practices to reduce Ni uptake by potentially contaminated matrices.

## Introduction

Food, specifically vegetables, represents a major source of nickel (Ni) exposure^1^. Environmental contamination and certain cultural practices can increase Ni amount in vegetables, posing significant risk for human health^2,3^. Nickel is a ubiquitous trace element occurring in water, soil, air and in the biosphere. This element is essential for several plants, microorganisms, and vertebrates^4^.

Tomato (*Solanum lycopersicum* L.) is a key vegetable worldwide, belonging to the Solanaceae family, the third most important commercial crop family from an economic point of view. This species has been used for a long time as a model plant in studies on disease response, genetics, and fruit ripening^5^. The fruit is produced for fresh consumption and processed products (e.g., tomato sauce, tomato paste, etc.) and is naturally rich in Ni, potentially affecting human health.

### Nickel in soil and food

According to the European Directive^6^ the limit values for Ni concentration in agricultural soils are 75 mg kg^−1^ (Dry Weight) at pH 7 and 30 mg kg^−1^ (Dry Weight) at pH 6, considering the higher bioavailability of most metals at lower pHs^6^.

The average Ni content in natural soils ranges between 13 and 37 mg kg^−1^, but significantly higher contents > 1000 mg kg^−1^ occur in ultramafic soils^7^, which systematically exceed, up to one order of magnitude, the threshold values laid down by governments and environmental agencies (e.g.^8^). Similarly, Ni content in agricultural soils generally does not exceed 100 mg kg^−1^^9,10^ but it can reach thousands of mg kg^−1^ in areas with ultramafic bedrocks^8,11,12^. Besides, agricultural soils intake of Ni may derive from atmospheric fallout, superficial and underground waters, and direct anthropogenic inputs^13^. Among these, atmospheric fallout represents an important and widespread input of Ni to soils, also for remote areas, due to the worldwide increasing emission of Ni to the atmosphere^14^, mostly from coal and oil combustion^7^. Direct anthropogenic inputs to agricultural soils are primarily due to mineral fertilizers, pesticides, compost, sewage sludge, and manure^13^.

The mobility of Ni in soils is strictly controlled by organic matter, amorphous oxides (mainly Fe and Mn oxides) and clay minerals. Oxides and clay minerals mostly scavenge Ni through sorption mechanisms and can release it to soil solution depending on pH variations^7^. Moreover, although the solubility of Ni in water is generally low (< 2 µg L^−1^), it can significantly increase in presence of dissolved organic compounds because it can form soluble complexes with organic ligands^15^, becoming potentially bioavailable. For instance, Ni mobility is quite high in acidic organic-rich soils, where fulvic and humic acids are formed by the decomposition of organic material^7,14^.

Ni plays and important part in plant physiology, as a component of the enzyme urease^16^, which participates in urea hydrolysis in several plant species^17^. In some plants, Ni is an essential micronutrient, promoting growth and development^18^. Ni toxicity levels are ∼ 10 μg [g dry weight (DW)]^−1^ in sensitive plant species^19^, and 50 μg g^−1^ DW in moderately tolerant species^20^. Ni phytotoxicity varies with the bioavailability of the metal and with the plant species^18^.

Currently, Ni is regulated under the European legislation^21^ regarding drinking waters with a threshold set at 20 μg L^−1^, instead of 70 μg L^−1^ as suggested by WHO^22^. Despite a specific legislation on Ni in food is missing, EFSA^23^ set a tolerable daily intake for body weight equal to 13 μg Ni kg^−1^. The EU commission raised awareness on Ni and adopted recommendation^24^ to monitor this metal in food in all Member States from 2016 to 2018 by sampling most representative foods, including tomatoes.

### Tomato crop and tomato allergy

Tomato crop is valued at 8–9G$, with international trade amounting to 4–4.5G$ annually^25^. Tomato is grown on about 5.8 Mha with a production of 243.9 MMt worldwide, 17 of which in EU^25^. Tomato was the top fruit produced in EU and, among vegetable crops of EU, it occupies the largest cultivated area, accounting for 11.4% of the total area used for vegetables^26^.

The significant rise in tomato consumption during the latest 20 years (e.g., Greece with 104 kg capita^−1^, Italy with 55 kg capita^−1^, Denmark with 30 kg capita^−1^^27^) is suspected to increase the health risks from high Ni uptake.

Ni allergy is common worldwide, and in EU it affects 10–15% of women^28^. The epidemiological data showed that 12.3–17.7% of the population is allergic to Ni^28^ and must follow a Ni-avoidance diet (e.g., Italy, Spain and Poland which have the highest incidence of Ni allergies). Low-Ni tomato products would be of great importance for these patients.

To date, seven tomato allergens have been identified and registered by the WHO/IUIS Nomenclature^29^. They are: profilin (Sola l 1), beta-fructofuranosidase (Sola l 2), fruit 9 kDa lipid transfer protein (9 k-LTP, Sola l 3), Bet v 1-like protein (Sola l 4), cyclophilin (Sola l 5), seed 7 kDa lipid transfer protein (7 k-LTP, Sola l 6) and a further 9 kDa lipid transfer protein from seeds (9 k-LTP, Sola l 7). Additional allergens, or putative allergens, not yet included in the WHO-IUIS nomenclature, such as 11 S globulin, chitinase, glucanase, peroxidase, polygalacturonase, pectin methylesterase, thaumatin-like protein (TLP) and vicilin have been reported and entered in the Allergome database^30^. Profilin, Bet v 1-like protein and LTP belong to allergen families that have been more widely studied compared to the others. Profilins and Bet v 1-like proteins are classified as class 2 food allergens, that are heat-labile, easily degradable by the gastrointestinal proteases and responsible for localized oral allergy symptoms (OAS)^31^. In contrast, LTP belongs to class 1 food allergens which are heat and protease-stable. They are clinically very relevant allergens because their ingestion, inhalation and contact can cause symptoms that may include all the clinical severity levels of allergic reactions: OAS, gastrointestinal symptoms, urticaria-angioedema syndrome, food-dependent exercise-induced anaphylaxis and even anaphylactic shock^32,33^. The plant LTP family includes two subfamilies, 9 k-LTP and 7 k-LTP, according to their molecular masses corresponding to 9 kDa and 7 kDa, respectively^34^. However, the allergic sensitization to 9 k-LTP is much more prevalent than that recorded for the smaller 7 k-LTP. In tomato, both these LTP have been found in seeds and the 9 kDa one has been recorded in the fruit^34,35^.

In most cases, tomato genotypes have been analysed from agronomic and technological point of view without considering Ni content and allergenic protein production that could increase the risk of allergies. Key geochemical processes that lead to limited Ni plant uptake in plant tissues at various growth stages can then be induced in field using different agricultural practices (irrigation, soil amendments, etc.).

This work aimed at assessing the *S. lycopersicum* response to Ni on the agronomic yield of tomatoes (i.e., plant biomass and fruit production) and the potential impact of Ni on the production of allergenic proteins (i.e., LTP, TLP, etc.).

## Results

### Soil Ni concentrations

The peat-sand mix (1:2 v/v) used as growing substrate was analysed by XRF and ICP-MS to determine the Ni concentration at the starting condition. The results evidenced that the Ni mean content of the substrate was 32 mg kg^−1^ (range 31–32 mg kg^−1^). This mean Ni content was assumed as the background value for the whole experiment. The separate analyses of the two components (peat and sand) evidenced that only the sandy fraction of the mixture was characterized by Ni content above the instrumental detection limits (range 46–47 mg kg^−1^) thus representing the only component of the substrate mix to contribute significantly to the initial Ni content of the substrate mix.

**Figure 1:**
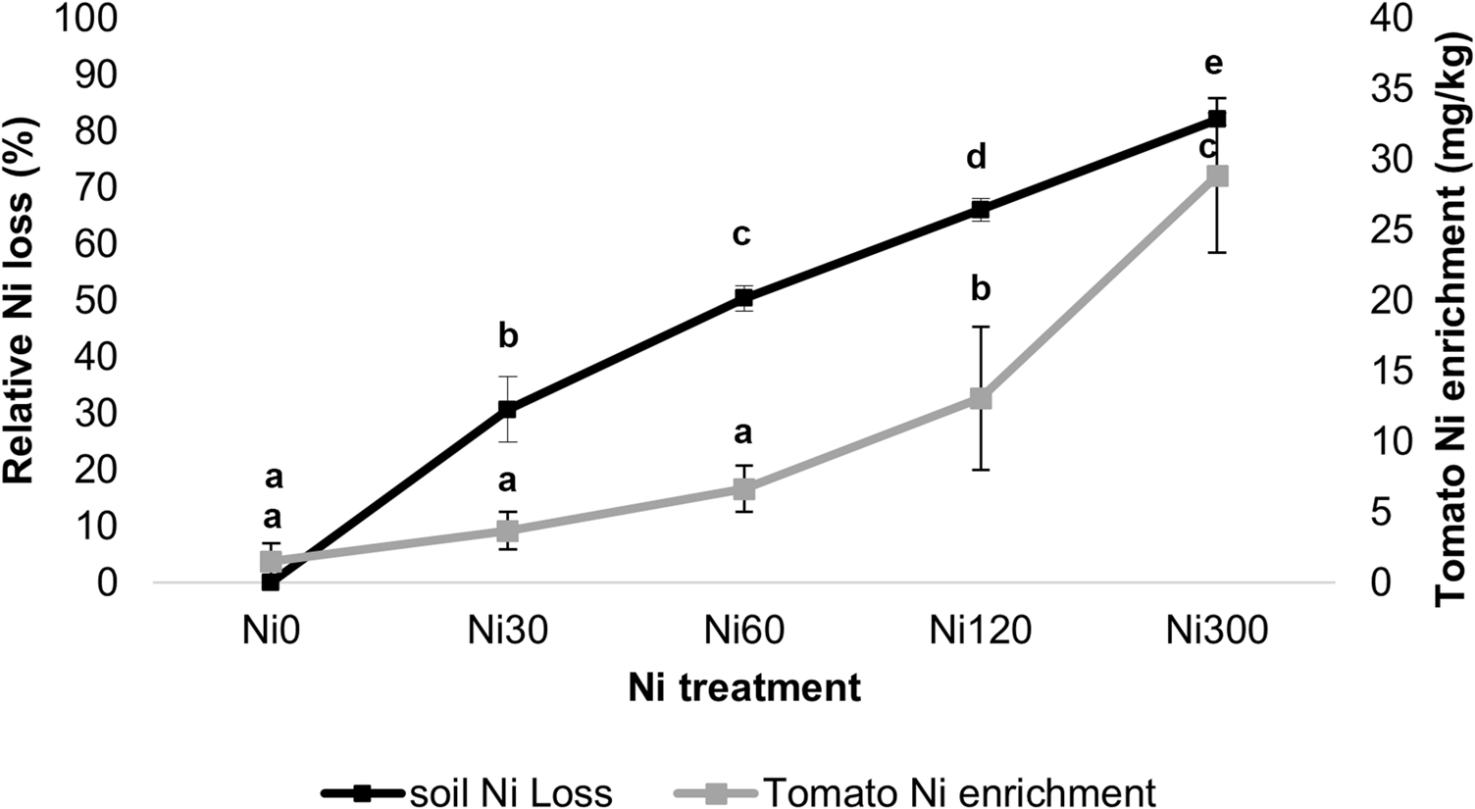
Relative Ni loss from soil (%) and Ni enrichment in tomato fruit (mg kg^−1^ DW), n = 10, each treatment. Data are average ± SD. Effective hypothesis decomposition, vertical bars denote 0.95 confidence intervals. Significant differences (obtained with Tukey’s post-hoc test) are marked with letter.

Considering the relative nickel loss, at the end of the experiment a significant amount of Ni, added as NiSO_4_·6H_2_O, was leached from soil. Relative Ni output increases in a linear way (Fig. 1), from Ni 30 to Ni 300. Nevertheless, the final Ni concentration in the soil always resulted into higher values than the starting value of the untreated substrate.

### Plant biomass development and fruit production in response to Ni

Considering plant productivity in terms of fruit produced (red, green, and total), biomass and fruit Ni accumulation, Spearman’s Rank Order Correlations (Table 1) does not highlight significant differences between Ni concentrations in fruits and fruit biomass or number.

**Table 1:** Spearman’s rank order correlations. MD pairwise deleted Marked correlations are significant at *P* < 0.05.

	Tot nRF	Tot nGF	Tot nF	Tot FW_RF	Tot DW_RF	Tot FW_GF	Tot DW_GF	Mean F weight	Mean Ni RF	Mean Ni GF	Mean Ni F
Tot. nRF	–	ns	0.65	0.74	0.70	ns	ns	ns	ns	ns	ns
Tot. nGF	ns	–	0.87	ns	ns	0.86	0.81	− 0.37	ns	ns	ns
Tot. nF	0.65	0.85	–	0.38	0.32	0.73	0.68	− 0.36	ns	ns	ns
Mean nF	0.65	0.85	ns	0.38	0.32	0.73	0.68	− 0.36	ns	ns	ns
Tot. FW_RF	0.74	ns	0.38	–	0.96	ns	ns	0.42	ns	ns	ns
Tot. DW_RF	0.70	ns	0.32	0.96	–	ns	ns	0.43	ns	ns	ns
Tot. FW_GF	ns	0.86	0.73	ns	ns	–	0.96	ns	ns	ns	ns
Tot. DW_GF	ns	0.81	0.68	ns	ns	0.96	–	ns	ns	ns	ns
Mean F weight	ns	− 0.37	− 0.36	0.42	0.43	ns	ns	–	ns	ns	ns
Mean Ni RF	ns	ns	ns	ns	ns	ns	ns	ns	–	0.89	0.96
Mean Ni GF	ns	ns	ns	ns	ns	ns	ns	ns	0.89	–	0.96
Mean Ni F	ns	ns	ns	ns	ns	ns	ns	ns	0.96	0.96	–

*Tot. nRF* total number of red fruits, *Tot. nGF* total number of green fruits, *Tot. nF* total number of fruits (red + green), *Mean nF* mean number of fruits produced, *Tot. FW_RF* total fresh weight of red fruits, *Tot. DW_RF* total dry weight of red fruits, *Tot. FW_GF* total fresh weight of green fruits, *Tot. DW_GF* total dry weight of green fruits, *Mean F weight* mean number of fruits weight, *Mean Ni RF* mean Ni concentrations in red fruit, *Mean Ni GF* mean Ni concentrations in green fruits, *Mean Ni F* mean Ni concentrations in fruits (red + green), *ns* not significant.

The Ni treatments do not correlate with tomato productivity while cultivars have negative significant correlations compared to the total number of fruits produced (ρ = − 0.36 *P* < 0.05; Table 2), but not compared to the fruit biomass. Green (G, unripe) and red (R, ripe) fruits do not show significant correlations compared to the other parameters.

**Table 2:** Spearman’s rank order correlations. MD pairwise deleted Marked correlations are significant at P < 0.05.

	Tot nRF	Tot nGF	Tot nF	Tot FW_RF	Tot DW_RF	Tot FW_GF	Tot DW_GF	Mean F weight	Mean Ni RF	Mean Ni GF	Mean Ni F
Ni treatment	ns	ns	ns	ns	ns	ns	ns	ns	0.84	0.80	0.82
Tomato cv	− 0.43	ns	− 0.36	ns	ns	ns	ns	ns	ns	ns	ns

*Tot. nRF* total number of red fruits, *Tot. nGF* total number of green fruits, *Tot. nF* total number of fruits (red + green), *Tot. FW_RF* total fresh weight of red fruits, *Tot. DW_RF* total dry weight of red fruits, *Tot. FW_GF* total fresh weight of green fruits, *Tot. DW_GF* total dry weight of green fruits, *Mean F weight* mean number of fruits weight, *Mean Ni RF* mean Ni concentrations in red fruit, *Mean Ni GF* mean Ni concentrations in green fruits, *Mean Ni F* mean Ni concentrations in fruits (red + green), *ns *not significant.

Since cv ‘Standard’ is more productive then cv ‘Ingrid’ in terms of fruits produced, productivity data were analysed grouped per cultivars obtaining the same results above mentioned.

The Kolmogorov–Smirnov two-sample test between controls and Ni treatments for Ni concentrations in fruit and fruit biomass (Table 3) showed that there is a significant difference between Ni in red, green and total fruit starting from Ni 60 only for cv ‘Standard’ and Ni 30 for cv ‘Ingrid’. However, there are no significant differences for fruit biomass and productivity between controls and Ni treatments.

**Table 3:** Kolmogorov–Smirnov two-sample test for comparison of controls (C) with the other Ni treatments (Ni 30, Ni 60; Ni 120; Ni 300 mg kg^−1^, respectively) with compared to mean μ and standard deviation σ considering tomato biomass parameters and Ni accumulation in fruit and the two cultivars used (‘Standard’ and ‘Ingrid’).

Parameters	'Standard'														'Ingrid'													
	C		Ni30			Ni60			Ni120			Ni300			C		Ni30			Ni60			Ni120			Ni300		
	μ	σ	μ	σ	P-level	μ	σ	P-level	μ	σ	P-level	μ	σ	P-level	μ	σ	μ	σ	P-level	μ	σ	P-level	μ	σ	P-level	μ	σ	P-level
Tot. nRF	4.4	3.0	3.4	1.7	P > 0.10	3.0	3.0	P > 0.10	4.0	3.0	P > 0.10	3.3	3.0	P > 0.10	2.8	1.6	1.0	1.0	P > 0.10	1.4	1.6	P > 0.10	1.8	1.6	P > 0.10	2.0	1.6	P > 0.10
Tot. nGF	4.0	3.8	2.8	5.2	P > 0.10	7.0	3.8	P > 0.10	4.3	3.8	P > 0.10	2.3	3.8	P > 0.10	2.2	1.9	1.7	2.1	P > 0.10	2.6	1.9	P > 0.10	1.3	1.9	P > 0.10	4.0	1.9	P > 0.10
Tot. nF	8.4	6.5	6.2	5.6	P > 0.10	10.0	6.5	P > 0.10	8.3	6.5	P > 0.10	5.5	6.5	P > 0.10	5.0	2.4	2.7	1.5	P > 0.10	4.0	2.4	P > 0.10	3.0	2.4	P > 0.10	6.0	2.4	P > 0.10
Tot. FW_RF	206.6	113.0	115.7	65.4	P > 0.10	65.3	113.0	P > 0.10	113.6	113.0	P > 0.10	118.8	113.0	P > 0.10	175.4	143.5	75.5	90.7	P > 0.10	49.1	143.5	P > 0.10	72.3	143.5	P > 0.10	112.1	143.5	P > 0.10
Tot. DW_RF	14.9	10.4	8.5	4.5	P > 0.10	5.0	10.4	P > 0.10	7.0	10.4	P > 0.10	9.2	10.4	P > 0.10	13.6	10.2	6.8	8.5	P > 0.10	3.8	10.2	P > 0.10	6.1	10.2	P > 0.10	9.9	10.2	P > 0.10
Tot. FW_GF	88.9	77.0	110.4	182.8	P > 0.10	215.2	77.0	P > 0.10	128.2	77.0	P > 0.10	64.3	77.0	P > 0.10	72.9	66.5	32.5	28.6	P > 0.10	88.5	66.5	P > 0.10	75.0	66.5	P > 0.10	83.5	66.5	P > 0.10
Tot. DW_GF	5.3	5.8	7.8	12.8	P > 0.10	12.7	5.8	P > 0.10	7.3	5.8	P > 0.10	3.9	5.8	P > 0.10	5.5	5.7	2.1	2.3	P > 0.10	5.8	5.7	P > 0.10	5.8	5.7	P > 0.10	6.3	5.7	P > 0.10
Mean F weight	42.1	14.0	41.7	19.3	P > 0.10	28.4	14.0	P > 0.10	40.4	14.0	P > 0.10	31.3	14.0	P > 0.10	64.2	37.0	46.0	33.1	P > 0.10	36.6	37.0	P > 0.10	78.6	37.0	P > 0.10	33.8	37.0	P > 0.10
Mean Ni RF	1.4	0.9	3.4	1.5	P > 0.10	**6.5**	**1.6**	**P > 0.05**	**17.0**	**14.5**	**P > 0.025**	**26.8**	**4.4**	**P > 0.025**	7.1	12.1	3.2	1.0	P > 0.10	4.9	2.1	P > 0.10	10.8	6.9	P > 0.10	25.5	7.7	P > 0.10
Mean Ni GF	1.1	0.7	1.7	1.2	P > 0.10	**8.0**	**2.6**	**P > 0.05**	**17.7**	**9.3**	**P > 0.025**	**31.4**	**17.0**	**P > 0.025**	8.4	16.5	7.4	9.3	P > 0.10	5.4	2.8	P > 0.10	16.5	9.6	P > 0.10	25.7	6.9	P > 0.10
Mean Ni F	1.2	0.5	2.6	0.9	P > 0.10	**7.3**	**0.5**	**P > 0.05**	**17.4**	**11.7**	**P > 0.025**	**29.1**	**10.2**	**P > 0.025**	7.7	14.3	5.3	5.0	P > 0.10	5.1	1.7	P > 0.10	13.7	8.1	P > 0.10	25.6	6.1	P > 0.10

P-levels are reported. Tomato parameters legend.

*Tot. nRF* total number of red fruits, *Tot. nGF* total number of green fruits, *Tot. nF* total number of fruits (red + green), *Tot. FW_RF* total fresh weight of red fruits, *Tot. DW_RF* total dry weight of red fruits, *Tot. FW_GF* total fresh weight of green fruits, *Tot. DW_GF* total dry weight of green fruits, *Mean F weight* mean number of fruits weight, *Mean Ni RF* mean Ni concentrations in red fruit, *Mean Ni GF* mean Ni concentrations in green fruits, *Mean Ni F* mean Ni concentrations in fruits (red + green), *ns* not significant.

Significant differences and significant P-levels marked in bold.

In addition, the Kolmogorov–Smirnov two-sample test to evaluate significant differences in plant biomass (root, stem, leaf, fruit DW and dry matter DM) between controls and Ni treatments (Table 4) revealed significant difference for ‘Standard’ from Ni 60 for stem (DW and DM), and from Ni 300 for leaf (DM). For ‘Ingrid’ revealed significant difference from Ni 60 and Ni 120 for stem DM, and from Ni 300 for leaf (DW and DM).

**Table 4:** Kolmogorov–Smirnov two-sample test for comparison of controls (C) with the other Ni treatments (Ni 30, Ni 60; Ni 120; Ni 300 mg kg^−1^, respectively) with compared to mean μ and standard deviation σ considering plant parameters and the two cultivars used ('Standard' and 'Ingrid').

Parameters	'Standard'														'Ingrid'													
	C		Ni30			Ni60			Ni120			Ni300			C		Ni30			Ni60			Ni120			Ni300		
	μ	σ	μ	σ	P-level	μ	σ	P-level	μ	σ	P-level	μ	σ	P-level	μ	σ	μ	σ	P-level	μ	σ	P-level	μ	σ	P-level	μ	σ	P-level
Leaf DW	42.0	7.8	38.0	5.6	P > 0.10	37.2	4.1	P > 0.10	34.0	9.5	P > 0.10	40.5	6.8	P > 0.10	43.0	5.7	33.3	14.9	P > 0.10	38.0	3.8	P > 0.10	29.8	15.0	P > 0.10	**30.2**	**2.2**	**P < 0.025**
Root DW	5.5	0.5	6.0	3.2	P > 0.10	6.7	1.9	P > 0.10	8.8	2.1	P < 0.10	5.6	3.9	P > 0.10	6.2	1.1	6.3	4.0	P > 0.10	9.8	4.4	P > 0.10	11.7	6.6	P < 0.10	7.1	2.1	P > 0.10
Stem DW	41.2	4.9	44.6	6.4	P > 0.10	**51.4**	**3.8**	**P < 0.025**	46.7	10.9	P > 0.10	46.8	5.3	P > 0.10	41.4	6.3	34.2	12.2	P > 0.10	49.0	6.5	P > 0.10	43.2	16.7	P > 0.10	41.9	3.5	P > 0.10
Tomato DW	20.2	15.8	16.3	10.8	P > 0.10	10.6	11.4	P > 0.10	20.2	15.8	P > 0.10	10.8	10.8	P > 0.10	19.0	13.9	5.3	8.9	P < 0.10	9.6	6.5	P > 0.10	9.5	6.5	P > 0.10	16.2	9.4	P > 0.10
Leaf DM	31.8	8.8	35.2	12.6	P > 0.10	27.7	4.4	P > 0.10	21.3	3.6	P < 0.10	**15.0**	**1.4**	**P < 0.025**	46.9	11.3	50.8	28.8	P > 0.10	37.2	11.1	P > 0.10	35.1	22.9	P < 0.10	**19.3**	**7.0**	**P < 0.025**
Stem DM	17.0	5.0	16.5	1.3	P > 0.10	19.2	1.2	P < 0.10	19.3	2.6	P > 0.10	15.0	1.6	P > 0.10	16.3	1.3	29.2	31.7	P > 0.10	**20.4**	**2.1**	**P < 0.025**	**32.5**	**27.4**	**P < 0.025**	19.3	2.7	P < 0.10
Root DM	17.5	1.7	16.8	3.8	P > 0.10	17.0	3.4	P > 0.10	19.6	2.3	P > 0.10	14.9	7.7	P < 0.10	15.8	1.0	14.8	6.5	P > 0.10	20.4	6.6	P > 0.10	19.7	4.3	P > 0.10	16.9	4.0	P > 0.10
Tomato DM	5.9	2.1	7.7	1.0	P > 0.10	5.0	3.5	P > 0.10	5.7	0.7	P > 0.10	3.1	3.1	P > 0.10	7.6	1.7	5.2	4.2	P > 0.10	7.6	1.5	P > 0.10	8.3	1.5	P > 0.10	8.5	0.6	P > 0.10

P-levels are reported.

Significant differences and significant P-levels marked in bold.

Parameters’ legend: dry-weight: DW; dry-matters: DM.

Summarizing, plant biomasses show significant differences in response to increasing Ni levels with clear evidence at Ni 300.

The same test between the two cultivars revealed significant differences between fruit and leaf DM, higher in ‘Standard’, supporting the evidence of a higher productivity of ‘Standard’ compared to ‘Ingrid’ (Table 5).

**Table 5:** Kolmogorov–Smirnov two-sample test for comparison between cultivar ‘Standard and ‘Ingrid’ compared to mean μ and standard deviation σ considering plant parameters.

Parameters	‘Standard’		‘Ingrid'		
	μ	σ	μ	σ	P-level
Tomato DW	15.5	12.8	11.9	10.0	P > 0.10
Leaf DW	38.3	7.0	34.9	10.4	P > 0.10
Stem DW	46.2	7.0	41.9	10.5	P > 0.10
Root DW	6.5	2.7	8.2	4.4	P < 0.10
Tomato DM	**6.0**	**2.3**	**7.5**	**2.2**	**P < 0.01**
Leaf DM	**26.2**	**10.0**	**37.9**	**20.0**	**P < 0.05**
Stem DM	17.4	3.0	23.6	18.3	P > 0.10
Root DM	17.2	4.2	17.5	5.0	P > 0.10

P-levels are reported. Significant differences and significant P-levels marked in bold. Parameters’ legend: dry-weight: DW; dry-matters: DM.

### Ni accumulation in tomato fruit

**Figure 2:**
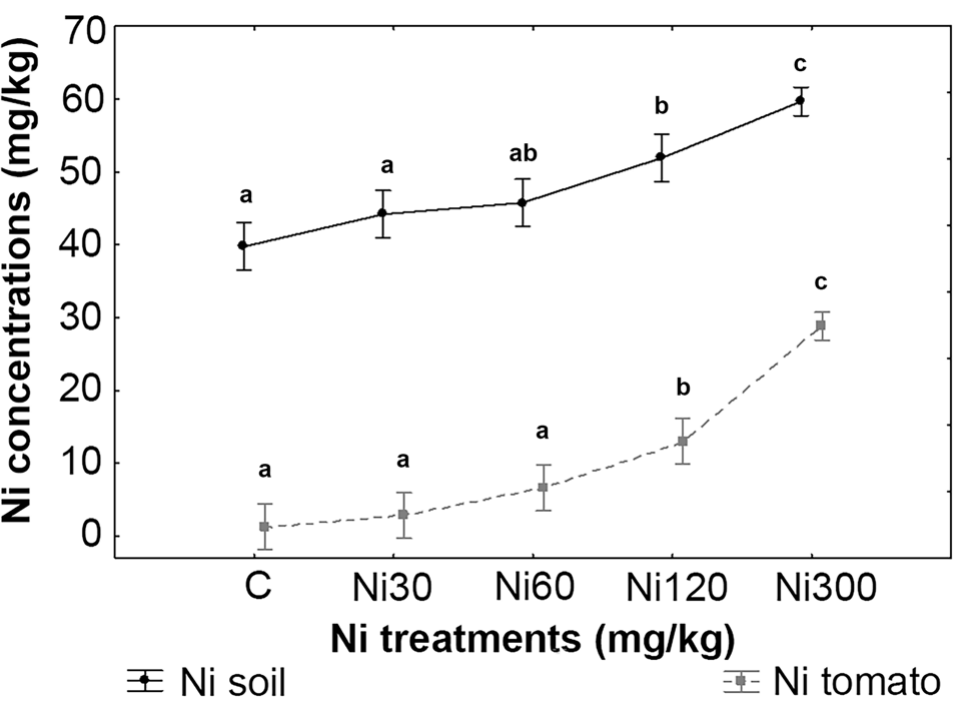
One-way ANOVA of Ni concentrations in soil and tomatoes at the end of the experiment compared to Ni treatments, n = 10, each treatment. Effective hypothesis decomposition, vertical bars denote 0.95 confidence intervals. Significant differences (obtained with Tukey’s post-hoc test) are marked with letter.

The one-way ANOVA performed on Ni treatments compared to Ni concentrations in soil and tomato at the end of the experiment (Fig. 2) highlighted significant differences from Ni 120 (*P* = 0.0002) and Ni 300 (*P* = 0.0001) compared to the control for soils and tomatoes with a marked significant difference also from Ni in tomatoes between Ni 120 (*P* = 0.0002) and Ni 300 (*P* = 0.0001).

The Pearson’s correlation between final concentrations of Ni in soil compared to Ni in tomatoes is highly significant (r = 0.83 *P* < 0.01).

In addition, the ratio between the Ni content in fruit compared to soil (F/S Ni) highlighted significant differences between control (F/S_0_ = 0.04 ± 0.03) and plant submitted to Ni 120 (F/S_120_ = 0.14 ± 0.03 *P* = 0.0002) and Ni 300 (F/S_300_ = 0.49 ± 0.10 *P* = 0.0001), respectively.

### Tomato allergens expressed under Ni stress

#### Protein extracts preparation from tomato samples

Extracts of the cultivar Standard, grown in five different conditions, namely at the Ni concentration of 0, 30, 60, 120 and 300 mg kg^−1^, were prepared and used for the analysis of protein and allergens content. Conversely, the samples of the cultivar Ingrid available for the same kind of characterization were three and were grown at the Ni concentration of 0, 30 and 300 mg kg^−1^.

**Figure 3:**
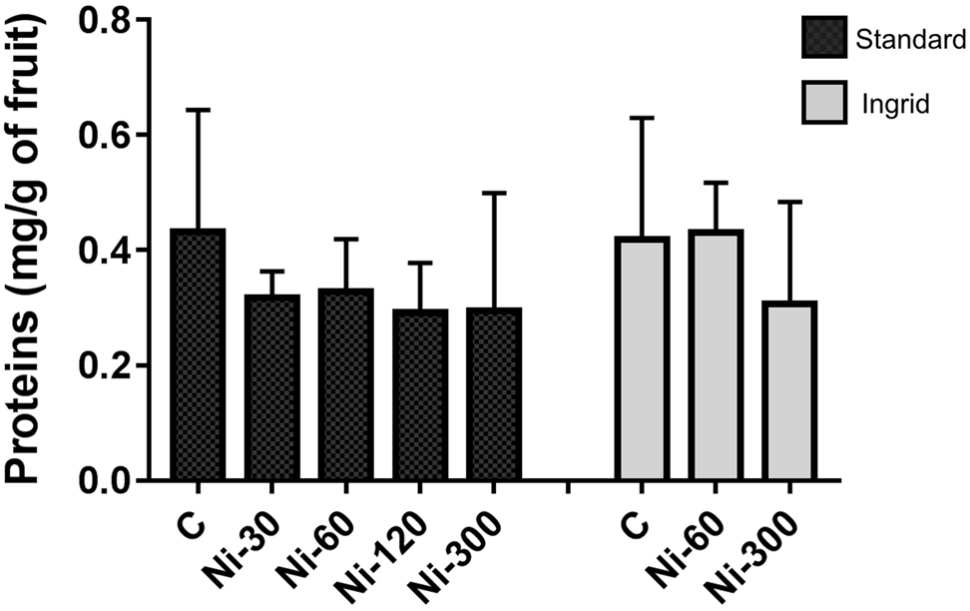
Protein concentration in tomato samples. Black and grey bars indicate the cultivars ‘Standard’ and ‘Ingrid’, respectively. Values are reported as mean with range from quadruplicate or triplicate measures. Only the value of ‘Ingrid’ cultivar Ni-60 derives from a duplicate.

Figure 3 shows the protein concentration in the total extracts, ranging from 0.07 to 0.51 mg g^−1^ of the fruit. The sample showing the highest concentration is C-’Ingrid’-G, followed by C-’Standard’-G and Ni 60-’Ingrid’-R. The Figure shows that the protein concentration found in both the controls of green tomato (C ‘Standard’-G and C ‘Ingrid’-G) is higher than that observed in the same samples after Ni-treatments, even in the one-way ANOVA with Tukey’s post-hoc test does not reveal significant differences.

#### Analysis of LTP and TLP content in tomato protein fractions using biochemical methods

**Figure 4:**
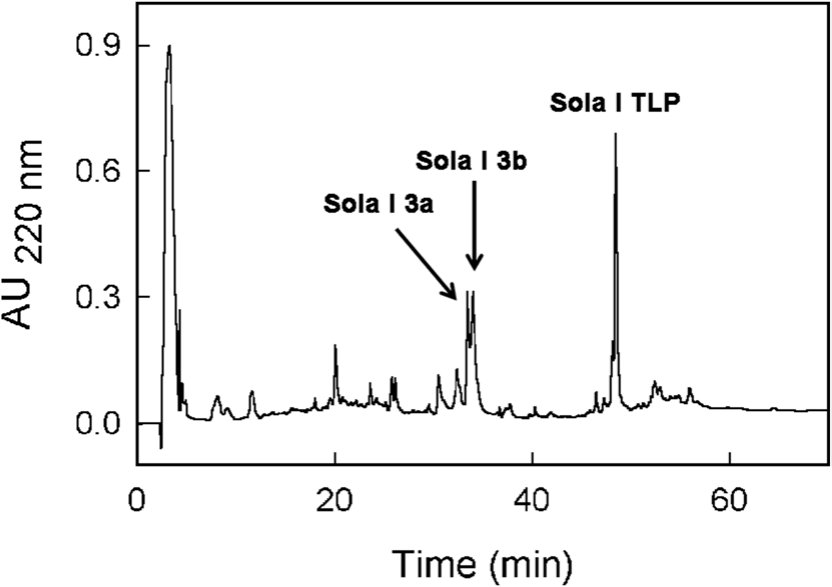
RP-HPLC profile of the fraction enriched in basic proteins obtained from mature (red) fruit of the control sample ‘Standard’. The peaks were manually collected and Sola l 3a, Sola l 3b and TLP were identified by N-terminal amino acid sequencing.

**Figure 5:**
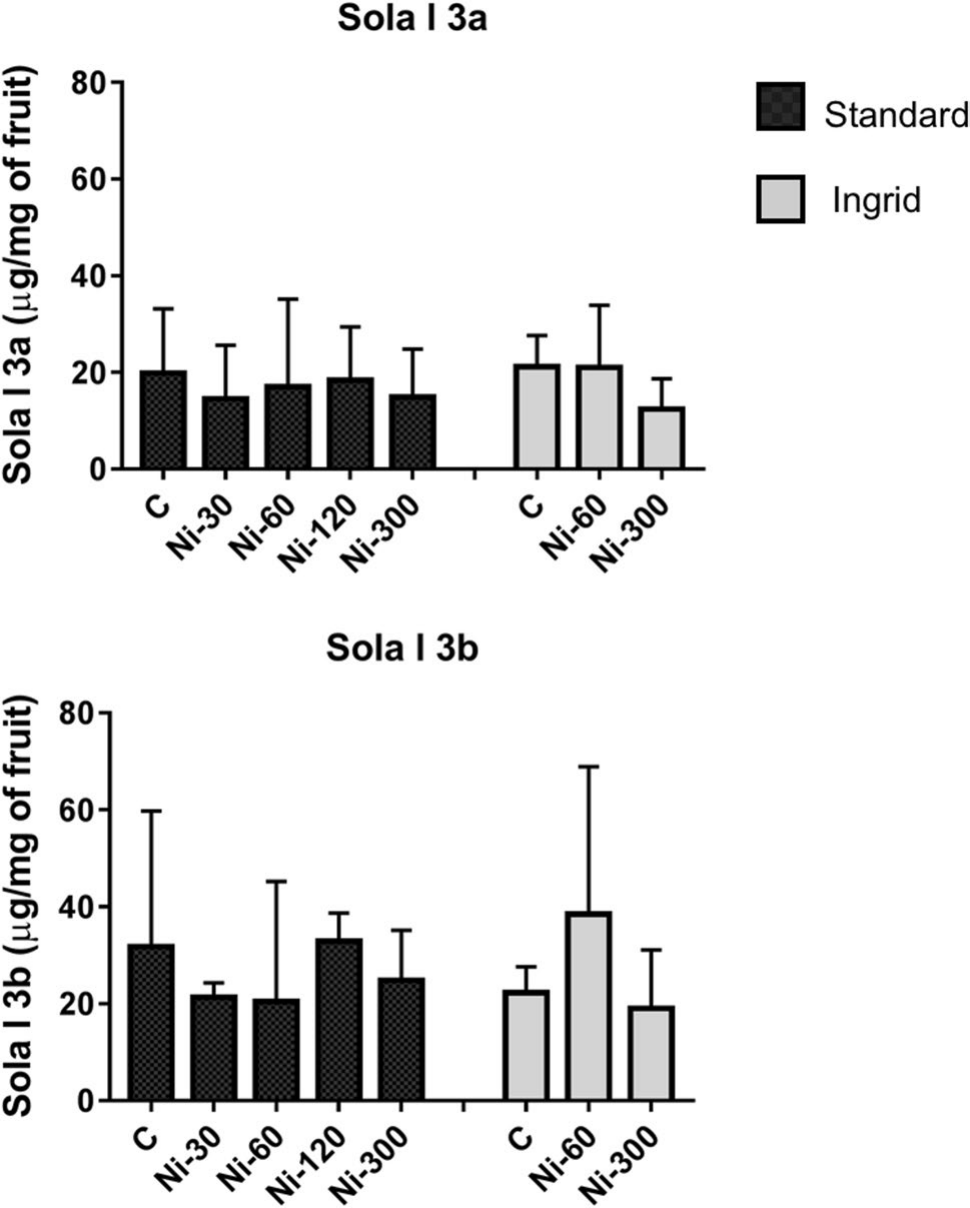
Concentration of Sola 3a and Sola l 3b in tomato samples. Black and grey bars indicate the cultivars ‘Standard’ and ‘Ingrid’, respectively. Values are reported as mean with range from quadruplicate or triplicate measures. Only the value of ‘Ingrid’ cultivar Ni-60 derives from a duplicate.

The analysis of RP-HPLC profile of tomato total extracts showed that the LTP detection and the estimation of its amount in the samples was not easy. This was especially due to the low concentration of this allergen compared to the other protein components. To overcome this issue, considering that LTP proteins are characterized by a basic isoelectric point, a fraction enriched in basic proteins was obtained from total extracts by separations with an anion exchanger resin. The samples were then concentrated as reported in the “Materials and methods” section and their protein profile was obtained by RP-HPLC. As an example, Fig. 4 shows the RP-HPLC profile of the fraction containing the basic proteins obtained from the extract of C ‘Standard’-R sample. The eluted peaks were collected and analysed by direct amino acid sequencing. Peaks eluted at 34.2 min and 34.8 min both provided the same N-terminal sequence, LSCGQVT. The similarity search against the UniProtKB database, with the BLASTP algorithm on the ExPASy server, allowed the identification of both the peaks as 9 kDa LTP, Sola l 3. At least two 9 k-LTP found in the UniProtKB database had the experimentally obtained N-terminal sequence (accession numbers A0A3Q7HZ96 and K4D1U9). They have been labelled as Sola l 3a and Sola l 3b (Fig. 4). Therefore, the detection in the RP-HPLC elution profile of more than one LTP peak indicates the presence of isoforms in the analysed samples. Figure 5 shows the amount of Sola l 3 estimated in the analysed tomato samples. It can be observed a certain variability of the Sola l 3 isoforms. However, it is not possible to observe any correlation between LTP concentration and the concentration of Ni applied in the treatments, nor significant differences with one-way ANOVA with Tukey’s post-hoc test are shown.

**Figure 6:**
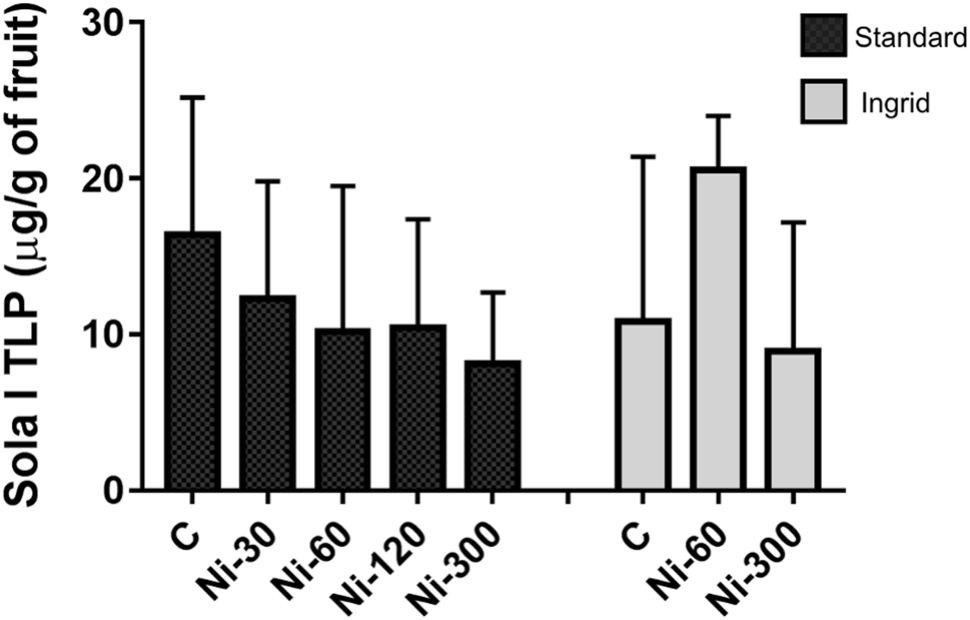
Thaumatin concentration in tomato samples. Black and grey bars indicate the cultivars ‘Standard’ and ‘Ingrid’, respectively. Values are reported as mean with range from quadruplicate or triplicate measures. Only the value of Ingrid cultivar Ni-60 derives from a duplicate.

The component eluted at 49.2 min was identified as TLP (Sola l TLP) by N-terminal amino acid sequencing that provided the following sequence ATKEVRNNCP (Accession number in UniProtKb P12670). Figure 6 shows a not significant decrease of TLP in the standard cultivar as a function of the increasing Ni concentration. The same effect is not observed in the ‘Ingrid’ cultivar.

#### Analysis of allergens content in the tomato samples by IgE inhibition tests

**Figure 7:**
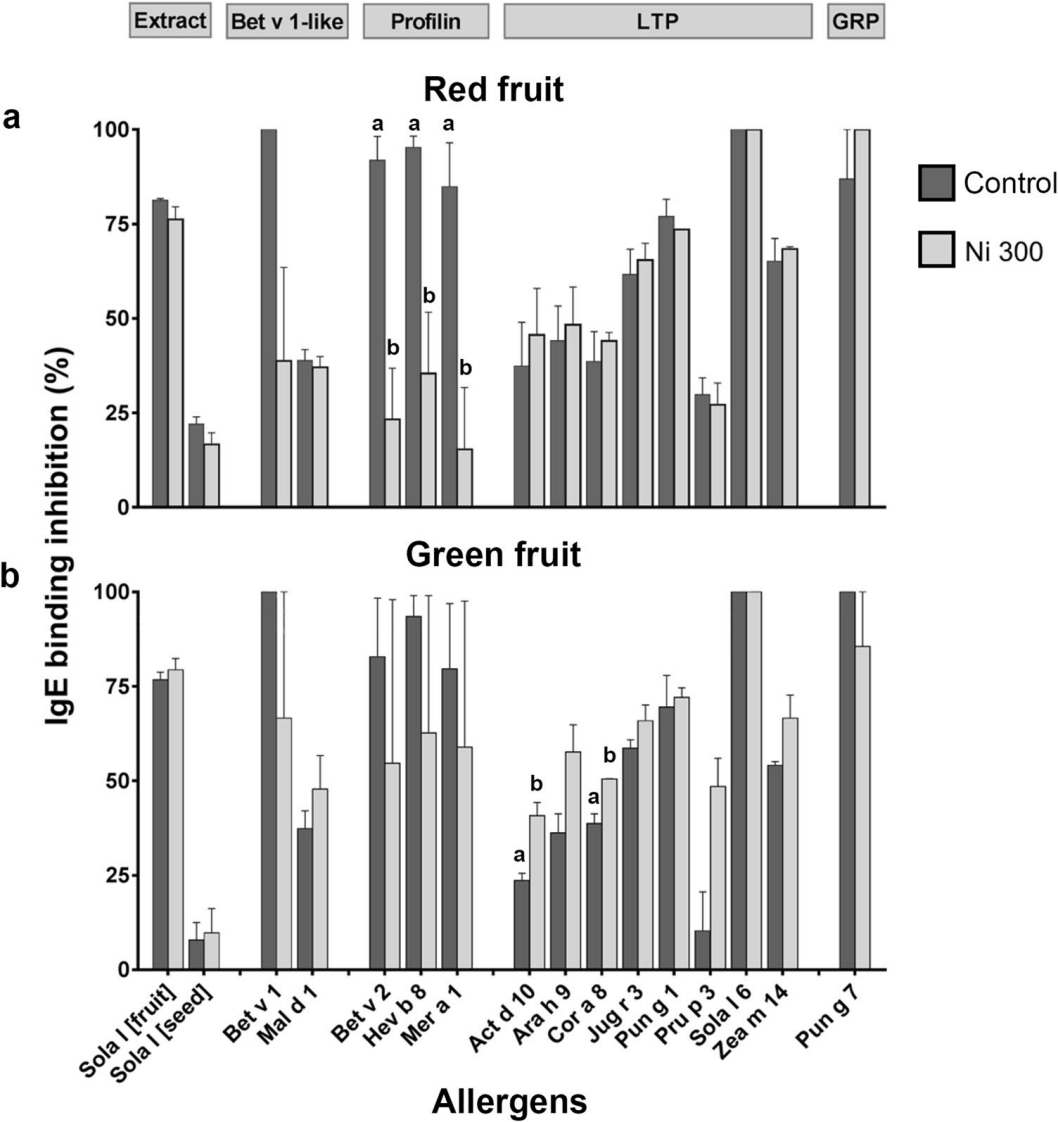
IgE Inhibition assays carried out with the tomato extracts of the ‘Standard’ cultivar using the FABER testing system with the SPHIAa method. Ripe—red (**a**) and unripe—green (**b**) tomatoes were probed with a mix of sera of patients sensitized to plant foods and used as providers of specific IgE. Untreated tomatoes are indicated with black columns, whereas Ni 300 treated samples are indicated with grey columns. Details of the analysed allergens are reported in Table 6. The values are duplicates reported as mean with range. Significant differences (obtained with Tukey’s post-hoc test) are marked with letters.

The allergens contained in the samples treated with Ni 300 were investigated by immunological tests and the results were compared with controls (untreated tomatoes). Two samples of Ni 300-treated red tomato and two samples of Ni 300-treated green tomato were analysed with the SPHIAa method^36^ on the FABER system^37^. Figure 7 shows the IgE-binding inhibition results recorded on some allergens spotted on the FABER biochip, namely the tomato fruit extract, tomato seed extract, Bet v 1 and a Bet v 1-like protein, three profilins and seven LTP (see Table 6 for details). In line with the observation that these tomato samples contained a very low number of seeds, results obtained show that the inhibition on the entire fruit extract is high, whereas lower values were recorded for the tomato seed extract.

**Table 6:** Details of allergens immobilized on the FABER biochip and probed with the tomato extracts using the SPHIAa test.

Protein family	Allergen	Source
n.a	Sola I [fruit]	Tomato (whole fruit)
	Sola I [seed]	Tomato seed
Bet v 1-like	Bet v 1	Birch pollen
	Mal d 1	Apple fruit
Profilin	Bet v 2	Birch pollen
	Hev b 8	Latex
	Mer a 1	Annual mercury pollen
LTP	Act d 10	Kiwifruit seed
	Ara h 9	Peanut seed
	Cor a 8	Hazelnut seed
	Jug r 3	Walnut seed
	Pun g 1	Pomegranate pulp
	Pru p 3	Peach fruit
	Sola I 6	Tomato seed
	Zea m 14	Corn seed
GRP	Pun g 7	Pomegranate pulp

The presence in the fractions of both, red (Fig. 7a) and green fruits (Fig. 7b), of tomato 7 k-LTP, Sola l 6, was indicated by the IgE-binding inhibition (100%) recorded on this allergen spotted on the FABER biochip. Inhibition with variable values was also observed on all the seven 9 k-LTP contained in the biochip. Nevertheless, the inhibitions produced by red tomato on these LTP appear independent of Ni treatment. Differently, compared with the control, results obtained with Ni-treated green tomato show a higher but not significant inhibition on the peach LTP, Pru p 3, and to a lower extent on other LTP, such as the peanut Ara h 9 and the maize Zea m 14, while the kiwifruit Act d 10 (*P* = 0.05) and the hazelnut Cor a 8 showed a significantly higher decrease (*P* = 0.05 and *P* = 0.04, respectively, with Tukey post-hoc test). All together, these results suggest a higher concentration of LTP Sola l 3 in Ni 300-treated green tomato compared to the control samples.

Ni-treated tomato inhibited Bet v 1 with a lower efficiency, compared to the untreated samples. This result suggest that nickel could induce a decrease of a tomato Bet v 1-like allergen. However, the result obtained on the Bet v 1-like allergen Mal d 1 did not produce the same result. Compared to the untreated tomato, red Ni 300-treated ones showed a significant lower IgE-binding inhibition on the three tested profilins (Bet v 2 *P* = 0.03; Hev b 8 *P* = 0.04; Merc a *P* = 0.04, with Tukey post-hoc test). This result is consistent with a significant reduction of profilin concentration in Ni-treated tomato. In addition to LTP, profilin and Bet v 1-like allergen, IgE-binding inhibition was detected also on the pomegranate GRP. Therefore, GRP may represent a new potential tomato allergen. The Ni-treatment seems to give only a weak effect on the GRP concentration in tomato.

### Proteins response to Ni in tomato samples

The Pearson’s correlation between fruit biomass and proteins expressed and final concentrations of Ni in soil and in tomatoes (Table 7) highlighted significant correlation between Ni in tomato and thaumatin (r = 0.40, *P* < 0.01) and Ni in soil and thaumatin (r = 0.51, *P* < 0.01).

**Table 7:** Pearson’s correlations comparing Ni soil and Ni tomato with fruit biomass and proteins expressed.

Parameters	Ni soil (mg kg^−1^)	Ni tomato (mg kg^−1^)
Fruit fresh weight (mg)	− 0.08	− 0.28
Extracts (mg ml^−1^)	− 0.38**	− 0.50**
Tot. protein (mg)	− 0.11	− 0.29*
Mg protein g fruit^−1^	− 0.33*	− 0.34*
LTP1 + LTP2 (µg g^−1^ of fruit)	− 0.24	− 0.20
LTP1 (µg g^−1^ of fruit)	− 0.32*	− 0.27
LTP2 (µg g^−1^ of fruit)	− 0.21	− 0.17
µg thaumatin g^−1^ of fruit	− 0.50**	− 0.40**

*Significant *P* < 0.05.

**Highly significant *P* < 0.01.

**Figure 8:**
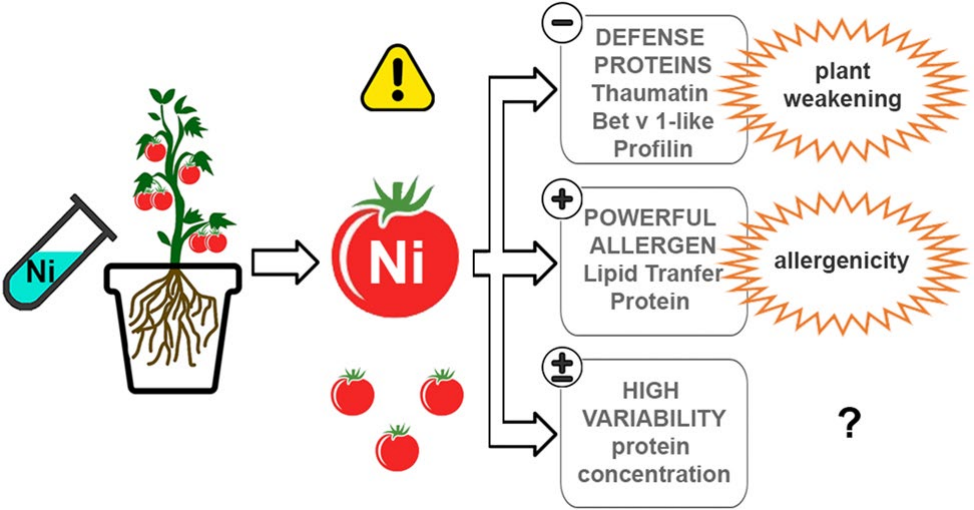
Schematic resume of the main results of the study.

The main results of the study were summarized in Fig. 8.

## Discussion

The evaluation of Ni uptake and storage in tomato fruits and related, induced, allergens expression is a key point to set up agricultural practices to limit Ni mobilization from soil.

The sandy fraction of the starting peat-sand mix, i.e., the Ni-bearing fraction of the growing substrate, behaved as an inert medium throughout the 240 days of the experiment. The relative loss of Ni increased linearly with increasing nickel sulphate hexahydrate additions, from Ni 30 through Ni 300 mg kg^−1^. This evidence suggests that most of the Ni in the experimental medium might be absorbed and translocated in the tomato plants or leached out with the excess water.

In addition, Ni is actively taken up and stored in fruit when soil concentration exceeded 120 mg kg^−1^. At the end of the experiment each soil replicate had the same amount of Ni, whereas the concentration stored in fruit is as twice as much as in lower concentration (i.e., 30, 60, 120 mg kg^−1^, respectively). Data from literature provided different evidence regarding this phenomenon sometimes highlighting a direct correlation of plant Ni as a function of the concentration of Ni in the soil^38,39^, as in our study. However, it is important to outline that, although growing condition and agricultural practices used are partly comparable, the soil used for our experiment is a peat-sand mixture (1:2 v/v) prepared in laboratory. Conversely, the cited experiments used natural soils such as dystrophic red Latosol (oxisol) with a sandy-clay texture^38,39^. In addition, the Ni salt added in some experiments^39^ is different and may affect the Ni bioavailable fraction in soil and the related mechanisms of Ni sequestration by roots.

Uptake from the cultivation soil is the main origin of metals and metalloids in edible parts of the plants. However, recent reports indicated that tomato has the general ability to store metals in fruits^40–44^ even if those values were sometimes lower than the allowable concentrations by FAO/WHO^45,46^, sometimes with high level of hazard that could be related with environmental pollution (use of fertilizers/pesticides/industrial wastewater irrigation) and air pollution (due to emissions from industries and vehicles)^47^ or local intensive agriculture practices, smelting, industries, and wastewater irrigation^42^.

Interestingly, a higher amount of leaf water is directly correlated with the highest Ni treatment, contrary to common response to heavy metal increase in tomato plants^48–51^.

Usually, controlled studies have shown that a high concentration of bioavailable Ni results in Ni toxicity, causing a measurable reduction in plant biomass^52,53^. The toxicity of metals, including Ni, on plant growth and water content^53–56^ is manifested as a decrease in transpiration through the decrease in stomatal conductance, which exert toxic effects on photosynthesis^4^, leading to a decrease in the photosynthetic rate. Interestingly, the highest level of water at the leaf level, when soil Ni reaches 300 mg kg^−1^, might suggest a specific osmotic reaction to counteract the Ni intake. This is known in other plants, specifically hyperaccumulators, where water intake represents a selected response to alleviate metal stress and Ni toxicity^57^.

The analysis of the total protein concentration in the different tomato samples revealed some high variations, even between the fruits that had received the same Ni treatment, thus suggesting that many factors can affect the protein content. For instance, the light exposure, mechanical stresses or phytopathogens attack can be different even for fruits of the same plant and could affect their physiology and protein concentration. However, the calculation of average amounts reduced the observed differences between the analysed fruits. In fact, aside from a few exceptions, most of the samples showed average values of about 0.3 mg of proteins per g of fruit. Nevertheless, no correlation between the protein concentration and Ni treatments was observed.

LTP is a relevant allergenic protein that can cause severe symptoms. It belongs to the group 14 of pathogenesis-related proteins (PR-14). The estimation of the LTP content using biochemical methodologies did not allow the detection of any correlation between the Ni treatments and the concentration of this allergen in tomato. Nevertheless, RP-HPLC chromatographic separations show a clear variation of the concentration of two 9 kDa LTP (Sola l 3) isoforms contained in the tomato samples. However, the analysis of Sola l 3 isoforms as separate components, or as sum of the two detected isoforms added together, do not show correlation with the Ni treatments. The lack of this kind of correlation was confirmed, at least for red tomato, following the analysis of the LTP content performed with immunological inhibition tests consisting in the competition of extract components with the allergenic molecules spotted on the FABER biochip^37^. Conversely, IgE-binding inhibition results obtained with the green tomato suggest a possible Ni effect producing a slight increase in LTP content. In fact, we detected a much higher inhibition on the peach LTP, Pru p 3, produced by the Ni-treated green tomato compared with the control. The inhibition on the other tested LTP was not always in line with this outcome. However, the heterogeneity of the inhibition results could be due to the different IgE-binding epitope panels associated to the individual tested LTP and to the multiple LTP isoforms contained in tomato. Anyway, the result obtained for Pru p 3, that is an allergen well-known as the most powerful one of this protein family^58^, prompt further future investigations to better understand the Ni effects on the concentration of this relevant allergen.

In the ‘Standard’ tomato cultivar, a decrease of thaumatin as a function of Ni increase was observed by biochemical methods. Tomato thaumatin is a potential allergen although its clinical relevance is not clear yet. It is a pathogenesis-related protein belonging to the group 5 (PR-5). Similar to other components of PR-5, tomato thaumatin is involved in the plant response to biotic and abiotic stresses and many factors can promote induction and regulation of its expression^59^. Using immunological methods, a significant decrease of the allergenic profilin Sola l 1 in the tomato samples treated with Ni 300 was observed. This effect is in line with the literature report describing the decrease of profilin in the leaves of basil grown on soil containing 500 mg kg^−1^ of Ni^60^. Therefore, the experimental results here described confirm the already reported Ni effect in decreasing the profilin concentration in the plant tissues. Profilins are actin-binding proteins, present in the cytoplasm of all eukaryotic cells where they play a key role in cell physiology. They are involved in processes such as organ development, wound healing, and defence from biotic attacks. Similarly, the Bet v 1-like proteins are pathogenesis-related molecules involved in host defence and their amount in Ni-treated tomato appears decreased. They belong to the group 10 (PR-10). Therefore, beyond the allergological implications, the observation that Ni can cause a decrease of the amount of thaumatin, Bet v 1-like protein and profilin, which are proteins involved in relevant physiological processes and host defence, suggests a possible effect of this chemical element in weakening the plant, which can become more sensitive to environmental biotic and abiotic attacks.

The immunological experiments also allowed the detection of a new potential allergen, belonging to the family of GRP, not yet reported in tomato. In fact, this component contained in the tomato fraction competed with the pomegranate GRP^61^ for the binding of specific IgE. GRP are proteins involved in plant development and their expression is up-regulated by the plant hormone gibberellin. Results obtained in this study suggest a low effect of Ni treatment on the concentration of this protein in tomato, but a confirmation by additional investigations is desirable.

In our case, the ability of transferring Ni from soil to fruit without affecting plant viability in terms of biomass, fruit yield, poses significant risks to consumers. In fact, without the evidence of plant suffering, these Ni-rich fruit can potentially be eaten by consumers.

The presence of Ni affects the concentration in plant tissues of protein components, including pathogenesis-related proteins and relevant allergens. However, what we can detect is the result of a complex combination of the effects of many factors where Ni is only one of them. The results here reported suggest that Ni can cause an increase of the allergenic LTP, Sola l 3 and a decrease of profilin (Sola l 1), Bet v 1-like protein (Sola l 4) and thaumatin-like protein. However, further studies are needed to try to understand the effects of different individual factors on each protein component of the tomato fruit.

The choice of low-Ni practices should be considered avoiding potentially contaminated matrices like wastewater or low-quality compost.

## Methods

### Substrate preparation

The peat-sand mix (1:2 v/v) was chosen as growing substrate; the substrate was autoclaved at 120 °C for 20 min, and oven-dried at 60 °C.

The final pH of substrate was measured with a pHmeter (pH 210, Hanna Instruments) by mixing an aliquot of soil with deionized water (ratio 1:3 w/v).

To determine the Water Holding Capacity (WHC), soil was transferred into a 10 cm Ø pot, 100 ml of water were added to 100 ml of dry soil placed in a funnel on a graduated cylinder. After waiting at least 1 h until the last drop, the WHC (%) was calculated based on the volume of water retained by the soil.

### Experimental design

Three-months-old plants of *Solanum lycopersicum* L. ‘Cuor di Bue Standard’ (n = 25) and ‘Cuor di Bue Ingrid’ grafted Beaufort (n = 25) were purchased from a plant nursery specialized in tomato cultivars, in compliance with national and international regulations, then transferred to the experimental greenhouse of the University of Genoa. Plants were transplanted to 18 cm Ø pot containing 2 kg of substrate previously described (one plant per pot, 5 replicates each concentration) adjusted to pH 6.00. At the beginning of the experiment, soil was homogeneously hydrated up to 70% WHC with solutions of sterile deionized water and metallic salt (NiSO_4_·6H_2_O) at increasing concentrations of Ni (0, 30, 60, 120, 300 mg kg^−1^, respectively) to evaluate the plant response. The Ni concentrations were chosen based on the threshold values for environmental Ni, as mandated by European laws^6^.

Pots were transferred to greenhouse and the plants were grown under semi-natural conditions at controlled temperature (21–27 °C) for 240 days and were irrigated two times a week with tap water to maintain 70%WHC. Each pot had a saucer to recover drainage water.

A water-soluble fertilizer Leader N–P–K (20−10−20 + MgO + Me) was dissolved in water at the concentration of 4 g l^−1^ and supplied for each pot once a week.

### Soil, plant, and fruit sampling

At the end of the experiment, the plants were subdivided into roots and shoots and were thoroughly rinsed, first with tap water and then with deionized water. Soil was firstly removed manually from the root system and then thoroughly rinsed away with tap and deionized water. Soil was further sieved to collect the remaining thin roots. The fresh biomasses of the different plant organs were weighed separately. Mature fruits were collected during the whole fruiting stage (start on June until November 2017) and weighed to evaluate fresh biomass.

Soils, roots, shoots, and fruits were then oven-dried at 60 °C for 48 h (soils and plants) and for 96 h (fruits). Dried samples were weighed to evaluate dry biomass and powdered using a ball mill (Retsch MM2000, Haan, Germany).

To evaluate plant water content, the Root:Shoot ratio for both fresh and dry biomass and the dry matter % (100*DW/FW) in root and shoot were calculated^57^.

### XRF and ICP-MS analyses

The chemical analyses for nickel were carried out on dried and porphyrized soils and plant samples by using the X-MET7500 Field Portable X-ray Fluorescence (FP-EDXRF) Spectrometers (Oxford Instruments) thanks to the collaboration with Geospectra S.r.l, Spin-Off of the University of Genoa. The FP-EDXRF instruments were calibrated using both fundamental parameters calibration determined by the manufacturer and site-specific calibration standards (SSCS) representative of the matrix analysed by FP-EDXRF.

Quantitative analyses were performed on 20 soil samples and 110 tomatoes, and the data quality level of the analyses was defined according to the Method 6200 of the US Environmental Protection Agency^62^.

Confirmatory analysis both for nickel in soils^62–64^ (EPA Method 6200, 3050B (SW-846), 6020B) and plant^65,66^ (UNI EN 13805:2014; UNI EN 15763:2010) samples were carried out at Leochimica S.r.l. laboratory (Zoppola, Pordenone, Italy) using inductively coupled plasma-mass spectrometry, (ICP-MS). The confirmatory analyses were performed on 5 soil samples (ICP-MS vs XRF ratio = 1:4) and 10 tomato plants (ICP-MS vs XRF ratio ≈ 1:11). The confirmatory analyses and the FPXRF data compared very well with regression correlation coefficients always exceeding 0.85 and relative standard deviations always ≤ 10%.

### Tomato allergens analyses

#### Preparation of protein extracts and separated fractions

Protein extracts were prepared from each tomato sample using an already reported procedure showing a good efficiency in the extraction of proteins from fruits^67^. Briefly, each tomato sample was homogenized after the addition of 0.5 M NaCl (1:1 w:v). After stirring for 2 h in an ice water bath, the sample was centrifuged at 10,400 × *g* for 1 h. The supernatant, representing the total protein extract, was collected, and dialyzed against 10 mM Tris–Cl, pH 8.0. A 1 ml aliquot of each total extract was stored at − 20 °C and later used to estimate the protein concentration.

The remaining amount of each extract was subjected to chromatographic separations to obtain a fraction enriched with basic molecules. To achieve this objective, each extract was loaded on a DE52 (Whatman, Brentford, UK) column, equilibrated in the same buffer used for the dialysis. The flow through was collected and dialyzed against 10 mM NaAc pH 5.0. To concentrate this basic protein fraction, it was loaded on a Sp-Sepharose (GE Healthcare Bio-Sciences AB, Uppsala, Sweden) column, equilibrated in the same buffer. The bound components were eluted with 0.5 M NaCl and the fractions containing the proteins were pooled. A volume corresponding to 1 g of fruit was separated by RP-HPLC, on a Vydac (Deerfield, IL, USA) C8 column (4.6 × 250 mm), using a Beckman System Gold apparatus (Fullerton, CA, USA). Elution was performed by a multistep linear gradient of eluent B (0.08% TFA in acetonitrile) in eluent A (0.1% TFA) at a flow rate of 1 ml min^−1^. The eluate was monitored at 220 and 280 nm. Peaks of interest were collected and analysed by N-terminal amino acid sequencing.

#### Estimation of the protein concentrations

The protein concentration of the total protein extract was determined by the Bradford method with the BIO-RAD Protein Assay (Biorad, Milan, Italy), using a calibration curve made with bovine serum albumin.

To estimate the amount of 9 k-LTP (Sola l 3) and thaumatin-like protein (Sola l TLP) contained in each sample (expressed as μg of protein per g of fresh tomato), the area of the RP-HPLC peaks was calculated and compared with that of peaks obtained with a known amount of tomato 9 k-LTP and TLP, respectively.

#### Amino acid sequencing

Proteins collected from RP-HPLC were concentrated with a centrifugal vacuum concentrator (Savant Speedvac Plus SC110 A, Ramsey, Minnesota, USA). Next, a protein amount corresponding to about 200 mAU at 220 nm was subjected to automated direct sequencing of the N-terminal region with a Protein Sequencer PPSQ–33B (Shimadzu Corporation, Tokyo, Japan).

#### IgE inhibition experiments with the SPHIAa assay on the FABER testing system

FABER (ADL S.r.l., Latina, Italy) is a multiplex in vitro serological test that allows the detection of IgE antibodies specifically recognizing allergens spotted on a biochip^68,69^. The FABER version used to perform this study (FABER 244-122-122) bears 244 allergenic preparations, namely 122 purified allergens and 122 multiple protein allergenic extracts. To obtain information on a possible variation of allergen content in the tomato samples treated with Ni 300 mg kg^−1^, compared to the untreated ones, the SPHIAa assay^36,37^ was used with some modifications. Briefly, characterized sera of patients sensitized to different plant food allergens, including those containing IgE recognizing the tomato 7 k-LTP (Sola l 6) and other plant allergens, such as 9 k-LTP, profilins, Bet v 1-like proteins and gibberellin regulated protein (GRP), were selected and pooled. Next, 0.12 ml of the sera pool were co-incubated with 0.12 ml of a solution containing 0.1 mg of the tomato extract fraction enriched in basic proteins. The IgE binding inhibition was evaluated by running the FABER test and recording the residual IgE binding on the allergens spotted on the biochip. Reference values for the lack of IgE binding inhibition were obtained by running control samples where the allergen solution was substituted with buffer only. The inhibition values were calculated in real time by a specific procedure developed within the InterAll software (version 5.0, ADL s.r.l.).

#### Patients’ sera

Sera used in this study were selected among those stored in the serum bank of ADL. These are residual sera deriving from venous blood sampling made for the routine allergy diagnosis by FABER test^31,68^. The features of each serum, in terms of content of IgE antibodies able to recognize and bind specific individual allergens (specific IgE) spotted on the FABER biochip, are recorded in the InterAll databank (version 5.0, Allergy Data Laboratories). Sera were selected based on the specific IgE content. The chosen ones were free of IgE recognizing cross-reactive carbohydrate determinants (CCDs). In fact, they were tested negative against CCD-bearing proteins used as markers, namely bromelain from *Ananas comosus* and peroxidase from *Armoracia rusticana*.

In the SPHIAa experiments, IgE is used as a probe to detect the presence of structural determinants, that is the epitopes of the proteins (purified or in mixture) under investigation. Therefore, the selection of sera was independent of the clinical history and/or symptoms of patients. For the SPHIAa assay, a pool of four sera able to recognize relevant plant food allergens was prepared. They contained IgE recognizing allergens such as LTP, profilin, Bet v 1-like proteins, GRP and thaumatin-like protein. The final dilution of each individual serum co-incubated with the tomato extract sample was 1:8.

All patients gave their informed consent to the use of their clinical data for research purposes in an anonymous form. In view of the purely comparative nature of this study, along with the fact that all venous blood samplings were part of routine clinical practice and that a residual part of the routine sample was used for inhibition experiments, a formal approval by the Ethical Committee was not necessary.

### Data analysis

The statistical analyses were performed with Statistica 8.0 (Statsoft Inc.) software. Results below the detection limits are presented as zero and were used as such in the calculations.

Nonparametric tests were used to avoid data transformation. Normality of parameters were evaluated with the Shapiro–Wilk test. Correlations between variables were analysed using Spearman’s Rank Order Correlations coefficient (ρ) using *P* < 0.05 to indicate statistical significance, since most of data exhibit a non-normal statistical distribution.

The Kolmogorov–Smirnov two-sample test was used to evaluate differences between control and Ni treatments.

Parametric analyses were used for Ni in fruit and soil compared to allergenic proteins’ production.

The dry matter (DM%), defined as the fruit dry weight (DW)/fruit fresh weight (FW)*100^70–72^ is also calculated for plant organs to evaluate the overall plant biomass production in response to an abiotic stress as in the case of soil nickel.

## Abbreviations

DM: Dry matter
DW: Dry weight
FABER: P-Friendly allergen nano bead array
FP-EDXRF: Field portable X-ray fluorescence
FW: Fresh weight
ICP-MS: Inductively coupled plasma-mass spectrometry
9 k-LTP: Sola l 7, 9 kDa lipid transfer protein from seeds
RP-HPLC: Reversed phase high-pressure liquid chromatography
Sola l 1: Profiling
Sola l 2: Beta-fructofuranosidase
9 k-LTP: Sola l 3, fruit 9 kDa lipid transfer protein
Sola l 4: Bet v 1-like protein
Sola l 5: Cyclophilin
7 k-LTP: Sola l 6, seed 7 kDa lipid transfer protein
SPHIAa: IgE Single Point Highest Inhibition Achievable assay
SSCS: Site-specific calibration standards
TLP: Thaumatin-like protein
TFA: Trifluoroacetic acid
WHC: Water holding capacity
